# Two different STAT1 gain-of-function mutations lead to diverse IFN-γ-mediated gene expression

**DOI:** 10.1038/s41525-018-0063-6

**Published:** 2018-08-20

**Authors:** Adi Ovadia, Nigel Sharfe, Cynthia Hawkins, Suzanne Laughlin, Chaim M. Roifman

**Affiliations:** 1 Division of Immunology and Allergy, Department of Paediatrics, The Hospital for Sick Children and The University of Toronto, Toronto, ON Canada; 2 The Canadian Center for Primary Immunodeficiency and The Jeffrey Modell Research Laboratory for the Diagnosis of Primary Immunodeficiency, The Hospital for Sick Children and The University of Toronto, Toronto, ON Canada; 3 Department of Laboratory Medicine & Pathobiology, The Hospital for Sick Children, Toronto, ON Canada; 4 Department of Radiology, The Hospital for Sick Children, Toronto, ON Canada

## Abstract

Signal transducer and activator of transcription 1 (STAT1) regulates multiple biological processes downstream of a variety of cytokine receptors in many cell types. Heterozygous gain-of-function (GOF) mutations in STAT1 have been associated with a diverse phenotype encompassing chronic mucocutaneous candidiasis (CMCC) and declining immunity. There is no clear correlation between STAT1 domain-specific mutations and phenotype, and it remains unclear why GOF mutations in STAT1 result in such a wide spectrum of clinical presentations. To begin exploring this dilemma, we have studied the patterns of gene expression mediated by two different GOF mutations. Analysis of IFN-γ response elements using RNA microarrays in cells transfected with the rare H629Y mutant or the common R274G mutant showed distinct patterns of gene expression. We show here that the impact of GOF mutations in STAT1 is variant-specific. This difference in gene expression may explain the diversity in clinical manifestations experienced by these patients.

## Introduction

Signal transducer and activator of transcription 1 (STAT1) is a transcription factor that controls many biological effects downstream of the type I interferons as well as other cytokine receptors in many cell types.^1^

Monoallelic mutations in STAT1 have been identified in patients with a great diversity of clinical manifestations and immune lesions. Mutations have been predominately associated with chronic mucocutaneous candidiasis (CMCC)^2,3^ with or without a variety of autoimmune manifestations.^4,5^ STAT1 mutations have been also associated with a gradual decline in cellular and humoral immunity leading to fatal viral infections.^6^

Increased STAT1 phosphorylation and DNA binding found in most patients promoted the notion that a gain-of-function (GOF) mechanism underlies all these disorders. Yet, a common mechanism would not appear to explain the diversity of presentations.

We compared here a common mutation in STAT1 with a different mutation identified in a patient that had a novel clinical presentation.

## Results and discussion

Here we describe a female patient of English descent who is currently 30 years old. She was born preterm at 32 weeks of gestation and suffered necrotising enterocolitis soon after birth. Since the age of 1 year old, she suffered recurrent candida infections, pneumococcal chest infections, complicated by pneumatocele and bronchiectasis that eventually required left lower lobe resection and by 15 years old she was diagnosed with primary ovarian failure.

**Fig. 1:**
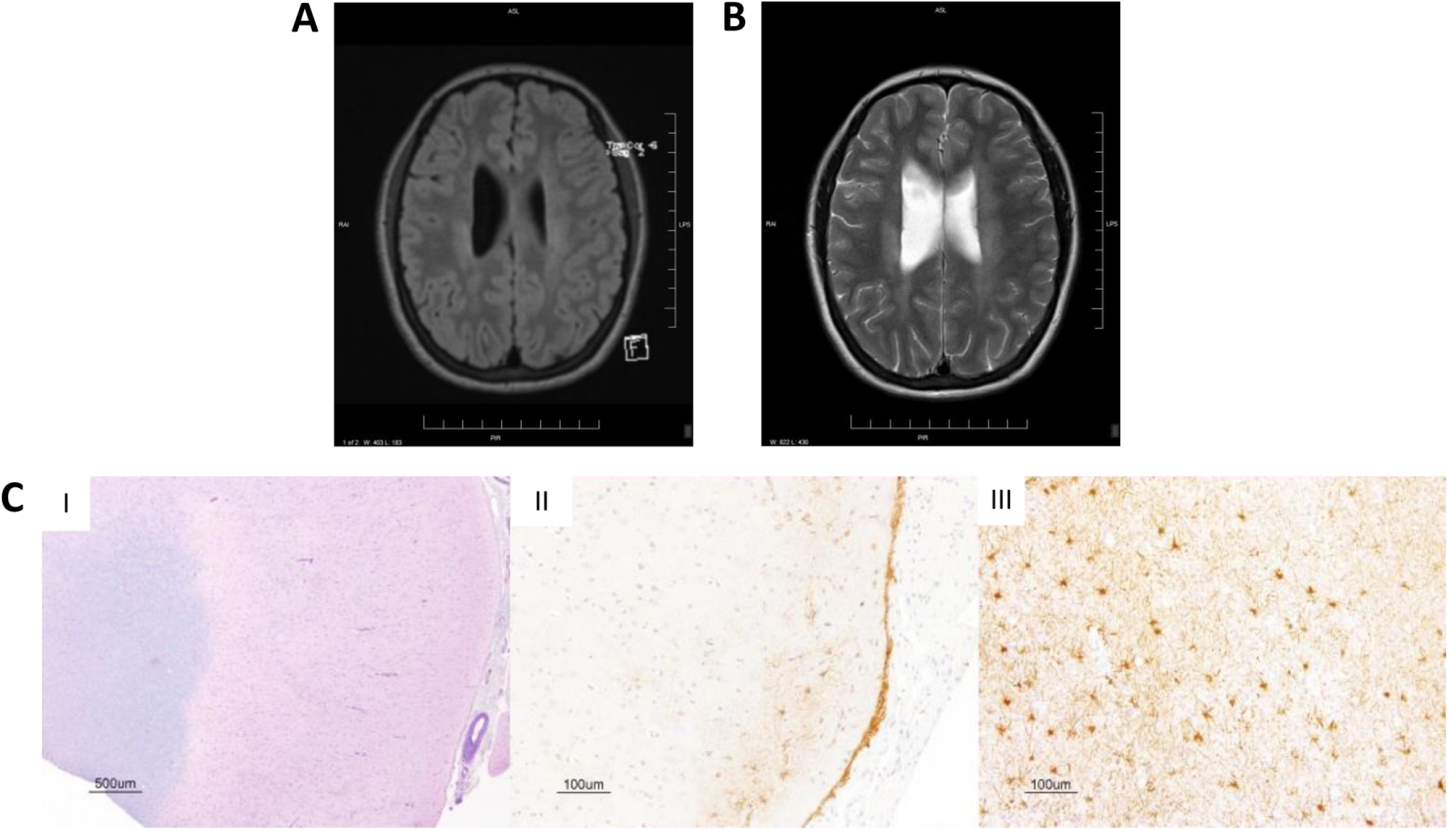
CNS changes in the patient. Brain magnetic resonance imaging demonstrating white matter periventricular lesions, as shown in the **a** fluid-attenuated inversion recovery (FLAIR) image, and **b** T2-weighted image. **c** Haematoxylin & eosin/luxol fast blue staining of the brain biopsy shows (I) normal architecture with preservation of grey-white differentiation. Immunohistochemical staining with glial fibrillary acidic protein demonstrates white matter and subpial gliosis (II, III). Images were taken at varying magnification with scale bars as shown

At 26 years, she had noticed gradual and progressive difficulty walking, right side weakness and spasticity, as well as dysarthria. Extensive neurological investigations, including visualised evoked potentials, brainstem auditory-evoked responses and somatosensory-evoked potentials were normal. Cerebrospinal fluid was negative for infectious agents and no oligoclonal banding was detected. Muscle biopsy showed no inflammation, necrosis or vascular changes. Magnetic resonance imaging of the brain and the spinal cord demonstrated diffuse abnormalities in the periventricular region and the white matter, with extensive wellerian degeneration in the brainstem affecting mainly the pyramidal tract (Fig. 1a, b). While brain aneurisms have been previously reported in association with GOF STAT1 mutations^2,4,5,7^ progressive neurodegenerative manifestations observed in this patient appear unique.

Evaluation of the immune system revealed normal numbers of circulating T, B and natural killer cells. In vitro proliferative responses to mitogens were normal but responses to antigens, including candida, cytomegalovirus (CMV), herpes simplex and herpes zoster were absent (Table 1). Serum immunoglobulin levels were normal but specific antibody responses to tetanus toxoid were non-protective.

**Table 1:** Immune evaluation of the patient

Parameter	Patient	Normal range/control
Lymphocyte immunophenotyping (cells/µL)		
CD3	1903	700–2100
CD4	1012	300–1400
CD8	784	200–900
CD56	348	90–600
CD19/20	1012	100–500
Responses to mitogens (stimulation index; P/C)		
PHA	358/500	Stim. index > 300 Patient/control > 50%
Responses to antigens (stimulation index; no. responses)		
Candida, CMV, zoster, simplex, tetanus, PPD	All < 20	Stim. index > 20 Total antigen responses > 2
Immunoglobulins		
IgG (g/L)	12.6	6.6–15.3
IgA (g/L)	1.1	0.54–4.17
IgM (g/L)	0.7	0.3–2.3
Specific antibodies		
Tetanus	0.02	>0.1
Measles/mumps/rubella	+ve/−ve/+ve	+ve/+ve/+ve
Isohemagglutinin	1:16	>1:32

**Fig. 2:**
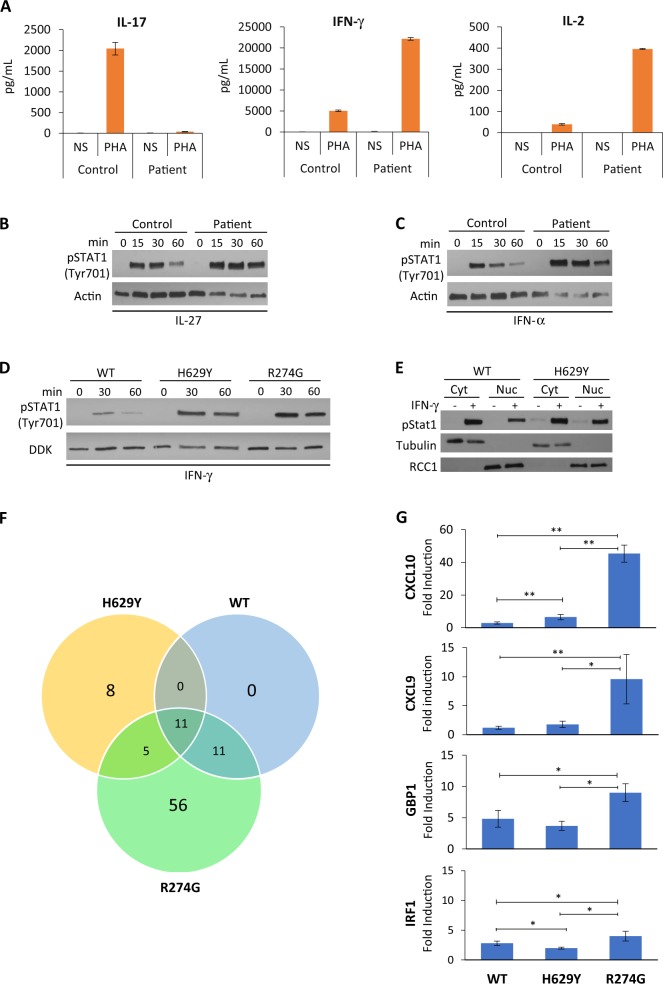
Abnormal STAT1 function and gene expression. **a** IL-17 (left panel), IFN-γ (middle panel) and IL-2 (right panel) secretion by PBMCs following phytohemagglutinin (PHA) stimulation for 48 h. Levels of indicated cytokines were determined by ELISA in triplicate samples. NS not stimulated. Western blot of activating STAT1 phosphorylation (anti-pTyr701) following stimulation with **b** IL-27 (10 µg/µL) and **c** IFN-α (8.5 ng/μL) in patient and control T-cell lysates. Anti-actin was used as a loading control. **d** STAT1 phosphorylation in STAT1 wild-type (WT), H629Y- and R274G-transfected U3A cells, following stimulation with IFN-γ (100 ng/mL). **e** Increase in phosphorylated STAT1 in the nuclear fraction of transfected U3A cells following IFN-γ stimulation. Anti-tubulin was used as the cytoplasmic marker and anti-RCC1 as the nuclear marker. Cyt cytoplasmic, Nuc nuclear. **f** Venn diagram of 1.5-fold change in IFN-γ-inducible genes. Diagram shows microarray analysis of upregulated genes following IFN-γ stimulation (100 ng/mL for 8 h) in U3A cells transfected with WT, H629Y or R274G STAT1. **g** U3A cells transfected as indicated with WT, H629Y or R274G STAT1 were stimulated for 8 h with IFN-γ (100 ng/mL) and mRNA levels of CXCL10, CXCL9, GBP1 and IRF1 were determined by q-PCR. Expression data, normalised to the levels of the house-keeping gene *GAPDH*, are presented as means and standard deviations from a total of 3–6 experiments. *P < 0.05 versus WT or R274G; **P < 0.01 versus WT or R274G (Student’s *t*-test)

Analysis of phytohemagglutinin (PHA)-induced cytokine secretion by patient T cells revealed virtually no interleukin (IL)-17 production as compared with control (Fig. 2a, left panel), while interferon-γ (IFN-γ) and IL-2 secretion were markedly increased when compared to control samples (Fig. 2a, middle and right panels).

Suspecting CMCC, we conducted Sanger sequencing for STAT1 and identified a de novo heterozygous c.1885C>T mutation in STAT1, predicting a histidine-to-tyrosine amino-acid change at position 629 (H629Y). No other immune-related gene aberration was detected using an immunodeficiency wide panel.

In order to understand why this patient has such a unique clinical pattern we studied gene expression patterns triggered by this mutation and compared it to the common mutant R274G, both designated GOF mutations. This common STAT1 mutation is typically associated with extensive candidiasis of the skin and mucous membranes and autoimmune endocrinopathy, infections and autoimmunity.

We first examined STAT1 phosphorylation as a determinant of GOF. We found increased STAT1 phosphorylation in H629Y-mutated patient T cells following IFN-α and IL-27 stimulation (Fig. 2b, c). Increased tyrosine phosphorylation was confirmed in STAT1-deficient U3A fibrosarcoma cells transfected with the H629Y mutant (Fig. 2d), comparable to phosphorylation in the R274G-transfected U3A cells, indicating that nuclear transit was not altered.

To further understand the effect of various GOF mutations, RNA microarrays were used to study downstream gene expression. RNA microarray analysis was performed on U3A cells transfected with either WT STAT1, the SH2 H629Y mutant or a ‘typical’ well-studied GOF mutant, R274G.^5^ The R274G mutant has been previously shown to increase STAT1 phosphorylation, enhance gamma-activated sequence DNA binding and induce an exaggerated upregulation of several genes in response to IFN-γ.^8^ Gene upregulation in this study was defined as a 1.5-fold increase over baseline of gene expression levels. The analysis revealed 24 IFN-γ-upregulated inducible genes in the H629Y mutant as compared with far greater (83 genes) triggered by the R274G mutation, while WT showed an increase of only 22 genes (Fig. 2f and Table 2). Eleven genes including *CXCL10*, *GBP1*, *GBP2*, *GBP4*, *GBP5*, *TAP1*, *PARP9*, *NMI*, *DTX3L*, *HLA-E* and *PSMB9* were commonly increased by WT STAT1 as well as both mutants. WT and R274G shared 11 other genes, which were highly expressed (*GBP3*, *IRF1*, *SP110*, *APOL6*, *AIM2*, *C1R*, *HAPLN3*, *LRRTM2*, *UBA7*, *TRIM69* and *IRF9*). Only 5 IFN-γ-inducible genes (*CXCL9*, *WARS*, *SERPING1*, *UBE2L6* and *TRIM21*) were commonly upregulated by H629Y and R274G. The H629Y mutant demonstrated uniquely high expression of TLR2, IFI44L, APOL3, TINAGL1, FZD10, OARD1, KLC2 and TRAV8-4, while the R274G mutant induced a rise in an additional 56 genes, which were not increased in WT nor in the H629Y mutant. These results indicated that different GOF mutations can affect IFN-γ-mediated responses by inducing diverse patterns of gene expression, with some shared while others private response genes.

**Table 2:** IFN-γ inducible genes upregulated >1.5-fold following IFN-γ stimulation in U3A cells

Variant	Gene
WT	*CXCL10*^a^, *GBP2*^a^, *GBP1*^a^, *GBP4*^a^, *GBP5*^a^, *TAP1*^a^, *PARP9*^a^, *NMI*^a^, *DTX3L*^a^, *HLA-E*^a^, *PSMB9*^a^, *GBP3*, *IRF1*, *SP110*, *APOL6*, *AIM2*, *C1R*, *HAPLN3*, *LRRTM2*, *UBA7*, *TRIM69*, *IRF9*
H629Y	*CXCL10*^a^, *GBP2*^a^, *GBP1*^a^, *GBP4*^a^, *GBP5*^a^, *TAP1*^a^, *PARP9*^a^, *NMI*^a^, *DTX3L*^a^, *HLA-E*^a^, *PSMB9*^a^, *CXCL9*, *WARS*, *SERPING1*, *UBE2L6*, *TRIM21*, *TLR2*^b^, *IFI44L*^b^, *APOL3*^b^, *TINAGL1*^b^, *FZD10*^b^, *OARD1*^b^, *KLC2*^b^, *TRAV8-4*^b^
R274G	*CXCL10*^a^, *GBP2*^a^, *GBP1*^a^, *GBP4*^a^, *GBP5*^a^, *TAP1*^a^, *PARP9*^a^, *NMI*^a^, *DTX3L*^a^, *HLA-E*^a^, *PSMB9*^a^, *CXCL9*, *WARS*, *SERPING1*, *UBE2L6*, *TRIM21*, *GBP3*, *IRF1*, *SP110*, *APOL6*, *AIM2*, *C1R*, *HAPLN3*, *LRRTM2*, *UBA7*, *TRIM69*, *IRF9*, *CXCL11*^c^, *UBD*^c^, *IDO1*^c^, *TNFSF10*^c^, *PARP14*^c^, *SAMD9L*^c^, *IL18BP*^c^, *ICAM1*^c^, *RTP4*^c^, *IFIT3*^c^, *CD74*^c^, *XAF1*^c^, *IFI30*^c^, *RSAD2*^c^, *STAT2*^c^,*TRIM22*^c^, *PRRT2*^c^, *CTSS*^c^, *PSMB8*^c^, *OAS2*^c^, *C1S*^c^, *CIITA*^c^, *PARP12*^c^, *PSMB10*^c^, *PDCD1LG2*^c^, *SLAMF8*^c^, *ACSL5*^c^, *IFIH1*^c^, *FLT3LG*^c^, *HLA-DRA*^c^, *IL32*^c^, *C4B*^c^, *GCH1*^c^, *SOCS1*^c^, *C4*^c^, *HLA-DOB*^c^, *MMP8*^c^, *TAP2*^c^, *HLA-A*^c^, *SPTLC3*^c^, *IFI35*^c^, *DDX58*^c^, *CD274*^c^, *TLR3*^c^, *IFIT2*^c^, *NREP*^c^, *NLRC5*^c^, *TAPBPL*^c^, *ERAP2*^c^, *RGCC*^c^, *ZC3HAV1*^c^, *BCL6*^c^, *XRN1*^c^, *HELZ2*^c^, *IRF2*^c^, *PML*^c^

WT wild type

^a^Gene expression commonly upregulated in WT, H629Y and R274G

^b^Gene expression unique to H629Y

^c^Gene expression unique to R274G

We next investigated whether the magnitude of response of gene amplification was similar among the different mutations for genes, which were increased in both mutants. For these experiments, quantitative PCR was used to compare responses to IFN-γ stimulation. As shown in Fig. 2g, the rise in CXCL10 in the R274G variant was far greater than the increase recorded in the H629Y mutant. Similarly, GBP1 and IRF1 expression was highest in the R274G variant. These results demonstrate that in addition to induction of a unique variant-specific set of genes, GOF variants also differ in the magnitude of the response.

Together, these findings indicate that STAT1 GOF mutants manifest in distinctively different patterns of gene expression with possible implication to the clinical phenotype. However, the relevance of this variable response to clinical manifestations cannot be deduced from this limited study of two mutations. A far larger study, including multiple different mutations as well as more extensive analysis of patients’ genome will likely be needed to address this important question.

## Methods

### Patient and blood samples

Data were compiled prospectively and retrospectively from medical records and were entered into the Canadian Centre for Primary Immunodeficiency Registry and Tissue Bank, which has been approved by the SickKids Research Ethics Board (protocol no. 1000005598). All patients provided written informed consent.

### Serum concentration of immunoglobulins and specific antibodies

Nephelometry was used to determine serum concentrations of immunoglobulins (IgG, IgA and IgM), and radioimmunoassay utilised to measure IgE levels (IgE PRIST kit, Pharmacia Diagnostics, Dorval, Canada).^6^

Serum antibody levels to tetanus toxoid and pneumococcus (pneumococcal capsular polysaccharide) were quantified by enzyme-linked immunosorbent assay (ELISA), as per the manufacturer’s instructions (Binding Site, Birmingham, UK). Serum antibodies to measles, mumps and rubella were also measured by ELISA (Euroimmune kits, Gross-Groenau, Germany). Serum isohemagglutinin titres are reported as the dilution at which macroscopic agglutination is observed (antiglobulin phase), and were determined by twofold serial dilution with erythrocytes.

### T-cell and B-cell proliferative responses

Lymphocyte proliferative responses to mitogens, including PHA, anti-CD3 and anti-CD28 antibodies, as well as a panel of recall antigens (candida, tetanus toxoid, herpes zoster, and CMV) were determined by thymidine incorporation, as reported previously.^9^ All assays were performed in triplicate and were compared with random normal controls.

### Sequencing analysis

Patient genomic DNA was amplified by PCR and sequenced for the *STAT1* gene. Primers were designed to amplify fragments up to 60 bp upstream and downstream of each transcript exon of the *STAT1* gene. PCR conditions consisted of 94 °C for 30 s, 55 °C for 30 s and 68 °C for 2 min, for a total of 35 cycles using Elongase (Invitrogen). PCR products were electrophoresed on 0.8% agarose gels. Each PCR fragment was subsequently cut and purified fragments were sequenced by DTCS Quick Kit on an automated sequencer (Beckman-Coulter CEQ 8000).

This mutation was confirmed in clinical lab setting whereby a primary immunodeficiency panel of 274 genes were analysed using next-generation sequencing methodology.

### Cytokine determinations

Ficoll-separated peripheral blood mononuclear cells from patients or controls were stimulated with PHA for 48 h or as indicated, and culture supernatants collected for analysis by ELISA.^10^ IL-2, IFN-γ and IL-17 cytokine analysis kits were obtained from R&D Systems (MN). Experiments were performed in triplicates.

### Cell lines and transfection

U3A cells (American Type Culture Collection) were grown at 37 °C in a humidified 5% CO_2_ atmosphere in complete growth media. STAT1-deficient U3A cells were cultured in Dulbecco’s modified Eagle’s medium (PAA Laboratories) supplemented with 10% foetal calf serum (Gibco). PLMV6-STAT1 was obtained from OriGene. STAT1 H629Y and R274G mutants were created by QuikChange II XL site-directed mutagenesis kit from Agilent according to the manufacturer’s recommendations. All mutations were confirmed by DNA sequencing. STAT1-deficient U3A cells were seeded into 10 cm plates and transfected using Lipofectamine 2000 (Life Technologies), according to the manufacturer’s instructions. U3A cells were serum-starved overnight prior to stimulation with 50 µg/mL IFN-γ for 30 min (phosphorylation) or 8 h (gene expression).

### Western blotting

Whole-cell lysates were prepared in RIPA buffer and analysed by western blotting.^6^ Cytoplasmic and nuclear fractions were prepared using the Pierce NE-PER subcellular fractionation kit (Thermo Scientific), as per the manufacturer’s instructions. Anti-STAT1 (SC-346), anti-tubulin and anti-RCC1 were purchased from Santa Cruz Biotechnology Inc. (Dallas, Tex). Anti-phospho-Stat1 (Tyr701) was purchased from Cell Signaling Technology (MA, USA). Anti-actin was purchased from Sigma (Darmstadt, Germany). All blots derive from the same experiment and were processed in parallel (Supplementary Figure 1).

### Microarray and quantitative real-time PCR

Total RNA was extracted from cultured cells with the RNeasy mini kit (QIAGEN). Microarray was performed by The Centre for Applied Genomics, The Hospital for Sick Children, Toronto, Canada. The Affymetrix Human Gene ST 2.0 array platform was used. For quantitative real-time PCR, 1 µg of total RNA was reverse-transcribed using SuperScript VILO cDNA synthesis kit (Invitrogen) according to the manufacturer’s instructions. The resulting cDNA amplified by PCR using the ABI 7500 Sequencer using SYBR select master mix (Life Technologies). Gene-specific primers for endogenous IFN-γ-inducible cDNAs (gbp1, cxcl10, cxcl9 and irf1) as well as for gapdh were used. In order to amplify fragments of about 200 bp in length, we used the following primer pairs: GBP1F, 5_-GGTCCAGTTGCTGAAAGAGC; GBP1R, 5_-TGACAGGAAGGCTCTGGTCT; CXCL10F, 5_-ATTTGCTGCCTTATCTTTCTG;CXCL10R; 5_-TCTCACCCTTCTTTTTCATTGTAG; CXCL9F, 5_-CCACCGAGATCCTTATCGAA; CXCL9R, 5_-CTAACCGACTTGGCTGCTTC; IRF1F, 5_-AGCTCAGCTGTGCGAGTGTA; IRF1R, 5_-TAGCTGCTGTGGTCATCAGG; GAPDHF; 5_-GAAGGTGAAGGTCGGAGTC; and GAPDHR, 5_-GAAGATGGTGATGGGATTTC. The relative expression of a transcript was normalised to the expression of GAPDH as determined for each sample. The ΔΔCt method was used to determine comparative relative expression levels. To compare gene expression data, Student’s test was used. *P* < 0.05 was considered statistically significant.

#### Data availability

The data that support the findings of this study are available from the corresponding author upon reasonable request.

## Electronic supplementary material

Supplementary Information

## Electronic supplementary material

**Supplementary information** accompanies the paper on the *npj Genomic Medicine* website (10.1038/s41525-018-0063-6).
