# Supplementary material for: Two different STAT1 gain-of-function mutations lead to diverse IFN-γ-mediated gene expression
Source: NPJ Genom Med. 2018 Aug 20;3:23. doi: 10.1038/s41525-018-0063-6 (PMC6102291; doi:10.1038/s41525-018-0063-6)
Supplement: Supplementary file 1 — Supplementary Information [file 41525_2018_63_MOESM1_ESM.pdf]

SUPPLEMENTARY FIGURE 1

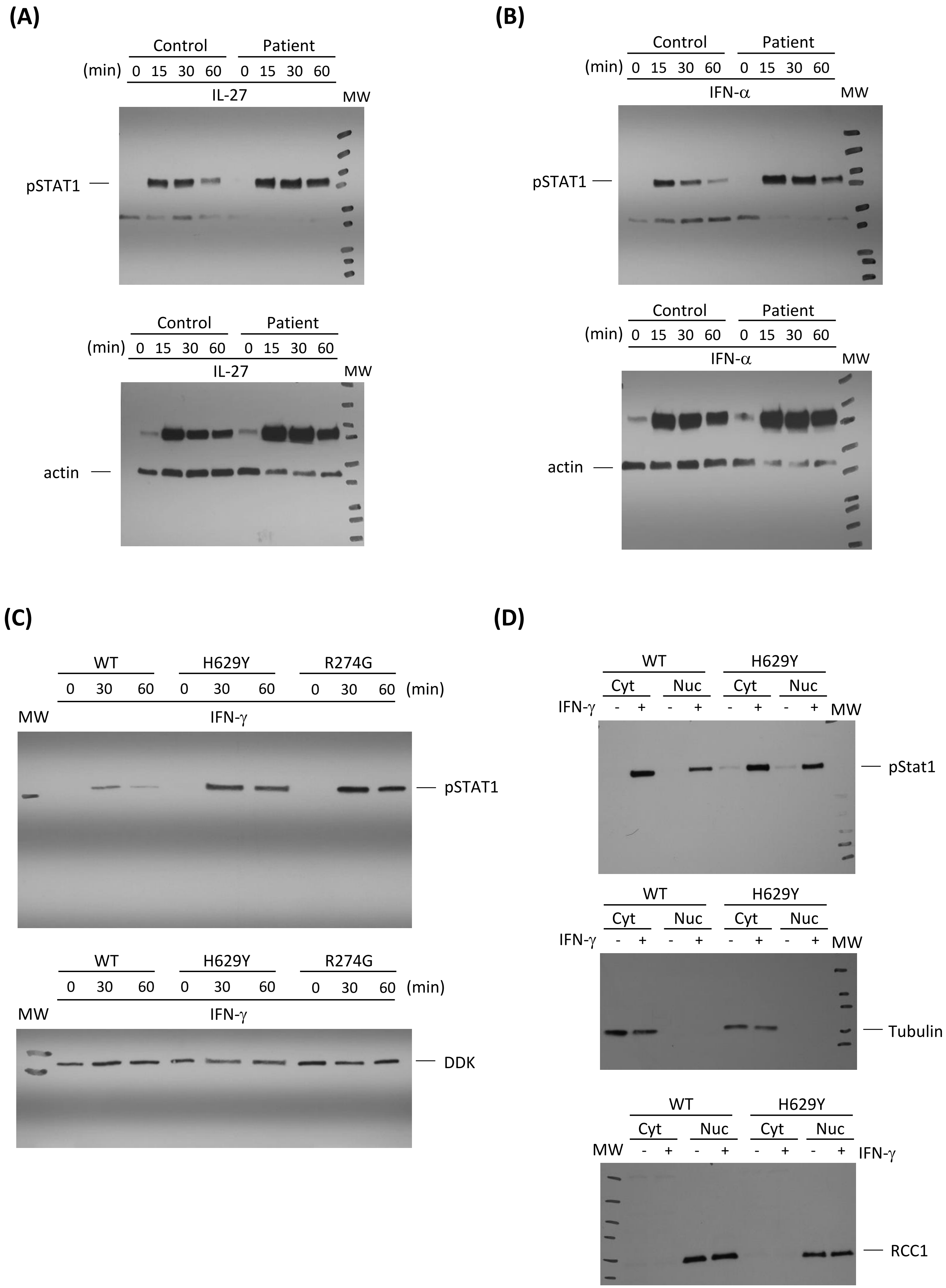

SUPPLEMENTARY FIGURE LEGENDS

Figure S1. Original western blots used to compile Figure 2B-E. Western blots demonstrating STAT1 phosphorylation (anti-pTyr701) following stimulation with **(A)** IL-27 (10 µg/µL; upper panel) and **(B)** IFN-α (8.5 ng/µL; upper panel) in patient and control T cell lysates. Anti-Actin was used as a loading control (lower panels). **(C)** STAT1 phosphorylation in STAT1 wild-type (WT), H629Y and R274G transfected U3A cells, following stimulation with IFN-γ (100 ng/mL). **(D)** Increase in phosphorylated STAT1 in the nuclear fraction of transfected U3A cells following IFN-γ stimulation. Anti-tubulin was used as the cytoplasmic marker and anti-RCC1 as the nuclear marker. Cyt, cytoplasmic; Nuc, Nuclear.
